# Very Low-Protein Diet (VLPD) Reduces Metabolic Acidosis in Subjects with Chronic Kidney Disease: The “Nutritional Light Signal” of the Renal Acid Load

**DOI:** 10.3390/nu9010069

**Published:** 2017-01-17

**Authors:** Biagio Raffaele Di Iorio, Lucia Di Micco, Stefania Marzocco, Emanuele De Simone, Antonietta De Blasio, Maria Luisa Sirico, Luca Nardone

**Affiliations:** 1 UOC di Nefrologia, A. Landolfi Hospital, Via Melito SNC, I-83029 Solofra, Avellino, Italy; luciadimicco@gmail.com (L.D.M.); deblasioantonella@libero.it (A.D.B.); ml_sirico@yahoo.it (M.L.S.); luca.nar@hotmail.it (L.N.); 2 Department of Pharmacy, School of Pharmacy, University of Salerno, Via Giovanni Paolo II 132, I-84084 Fisciano, Salerno, Italy; smarzocco@unisa.it; 3 UOC di Nefrologia, AORN San Giuseppe Moscati, I-83100 Avellino, Italy; ema.desimone@gmail.com

**Keywords:** Chronic Kidney Disease (CKD), Very Low-Protein Diet (VLPD), metabolic acidosis, nutritional light signal

## Abstract

Background: Metabolic acidosis is a common complication of chronic kidney disease; current guidelines recommend treatment with alkali if bicarbonate levels are lower than 22 mMol/L. In fact, recent studies have shown that an early administration of alkali reduces progression of CKD. The aim of the study is to evaluate the effect of fruit and vegetables to reduce the acid load in CKD. Methods: We conducted a case-control study in 146 patients who received sodium bicarbonate. Of these, 54 patients assumed very low-protein diet (VLPD) and 92 were controls (ratio 1:2). We calculated every three months the potential renal acid load (PRAL) and the net endogenous acid production (NEAP), inversely correlated with serum bicarbonate levels and representing the non-volatile acid load derived from nutrition. Un-paired *T*-test and Chi-square test were used to assess differences between study groups at baseline and study completion. Two-tailed probability values ≤0.05 were considered statistically significant. Results: At baseline, there were no statistical differences between the two groups regarding systolic blood pressure (SBP), diastolic blood pressure (DBP), protein and phosphate intake, urinary sodium, potassium, phosphate and urea nitrogen, NEAP, and PRAL. VLPD patients showed at 6 and 12 months a significant reduction of SBP (*p* < 0.0001), DBP (*p* < 0.001), plasma urea (*p* < 0.0001) protein intake (*p* < 0.0001), calcemia (*p* < 0.0001), phosphatemia (*p* < 0.0001), phosphate intake (*p* < 0.0001), urinary sodium (*p* < 0.0001), urinary potassium (*p* < 0.002), and urinary phosphate (*p* < 0.0001). NEAP and PRAL were significantly reduced in VLPD during follow-up. Conclusion: VLPD reduces intake of acids; nutritional therapy of CKD, that has always taken into consideration a lower protein, salt, and phosphate intake, should be adopted to correct metabolic acidosis, an important target in the treatment of CKD patients. We provide useful indications regarding acid load of food and drinks—the “acid load dietary traffic light”.

## 1. Introduction

Metabolic acidosis is a common complication of chronic kidney disease (CKD) when the glomerular filtration rate falls below 30–40 mL/min/1.73 m^2^ [1,2,3]. Current guidelines recommend treatment with alkali when bicarbonate levels are lower than 22 mMol/L [1] to prevent complications, such as insulin resistance [4,5], cardiovascular diseases and progression of CKD, among others [6,7].

A correction of metabolic acidosis can be achieved with pharmacological administration of oral alkali as sodium bicarbonate, but also with a diet rich of fruit and vegetables [8,9,10]. In addition, conservative therapy of CKD with low-protein content diets has been revised and reached the scientific rigor of nutritional therapy with the purpose of obtaining a reduction of sodium intake and hypertension [11,12], of phosphate intake and serum phosphate levels [13,14], of proteinuria [15,16], and a delay of dialysis initiation [17], through a reduced load of catabolites and a better metabolic control [17,18,19]. Hence, as fruit and plant proteins are able to reduce dietetic acid load [20,21,22], our aim is to evaluate whether a very low-protein diet (VLPD) reduces both the acid load in patients with CKD and the need of administrating oral sodium bicarbonate. 

## 2. Materials and Methods

**Figure 1:**
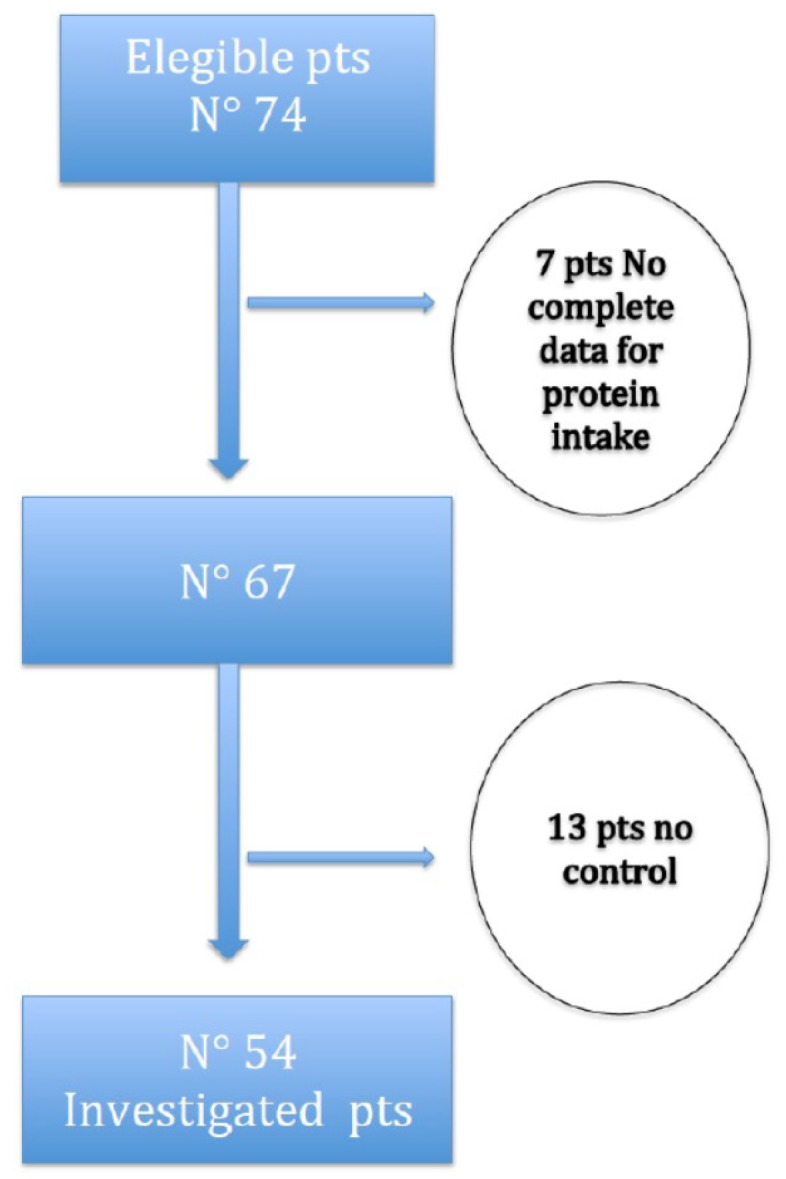
Flow chart of the study.

We conducted a case-control study in 146 patients participating in the UBI study—a multicentric, prospective, cohort, randomized, open-label and controlled study, registered in ClinicalTrials.gov (NCT01640119), in which patients were randomized to receive either oral sodium bicarbonate or placebo, in order to evaluate mortality and dialysis risk or doubling of creatinine levels [23]. All 146 patients were chosen from the experimental arm of the UBI study; therefore, they were all administered oral bicarbonate: 54 out of them assumed a VLPD and 92 case-controls assumed a control diet together with oral bicarbonate. The duration of follow-up was 12 months. We excluded diabetic subjects to avoid bias due to glycemic control complications. Figure 1 shows the study-flow diagram. Seventy-four subjects assuming VLPD encountered study criteria; 7 of them were excluded because of incompleteness of urinary data needed to calculate protein intake, and 13 because of lack of corresponding controls. Finally, among the included patients (54), only 38 of them had a case-control ratio of 1:2, while the other 16 subjects had a 1:1 ratio.

VLPD consisted of a vegetarian, high-energy diet, with a protein content of 0.3–0.4 g/Kg body weight (BW)/day supplemented with amino-acids and ketoacids in tablets (AlfaKappa^®^ Fresenius Kabi, Verona, Italia). The main nutritional differences between VLPD and control group diet were plant and animal proteins amount, phosphate and salt content (see Table 1).

During the course of CKD several metabolic alterations appear (hyperazotemia, metabolic acidosis, hyperpotassiemia, hyperphosphatemia, increase of parathyroid hormone (PTH) levels, intestinal dysbiosis), that are all positively influenced by a nutritional therapy using a VLPD. This diet is characterized by low-protein content with supplements of essential amino-acids and ketoanalogues (proteins used are roughly 90% of plant type), low phosphate content (300–450 mg in VLPD versus 1000–1200 mg in an usual diet), low salt content (80–95 mmol/day in VLPD versus 140–180 mmol/day in a usual diet), and low organic acids and saturated fats amount. The effects of VLPD in terms of reduction or normalization of urea levels in CKD are excellent, but the effects on other biochemical parameters are also worthy of interest [25].

Every 6 months, study participants performed the common biochemical blood tests and urine parameters (24 h urine urea, creatinine, phosphate, potassium, natrium and protein levels). Particular attention was paid to a correct 24 h urine collection, and all patients were trained in detail how to perform a correct procedure. 

Actual protein intake was evaluated according to the formula of Maroni et al.: daily protein intake (g/day) = (6.25 × urinary urea nitrogen mg/day) + (0.031 × body weight-kg) + urinary proteins g/day [26], using 24-h urinary urea and not dietetic interviews. Twenty-four hour urinary urea excretion together with urinary potassium and phosphate excretion were used to calculate the potential renal acid load (PRAL) and net endogenous acid production (NEAP) every 3 months. PRAL and NEAP, both inversely correlated with serum bicarbonate levels, represent the non-volatile acid load derived from nutrition, estimated by the production of non-volatile acids and bases produced during digestion based on known nutritional content [27]. PRAL was calculated by the following formula as described by Remer and Manz [28]: calculated PRAL (mEq/day) = (0.49 × protein intake, g/day) + (0.037 × phosphorus intake, mmol/day) − (0.021 × potassium intake, mmol/day) − ( 0.026 × magnesium, mmol/day) − (0.013 × calcium, mmol/day); NEAP was calculated by the formula described by Frassetto et al. [29]: NEAP (mEq/day) = (54.5 × protein intake (g/day)/potassium intake, (mmol/day)) − 10.2. Thus, protein intake contained in the two previous formulas was calculated with Maroni formula [26], and phosphorus and potassium intake were estimated from 24-h urinary phosphate and potassium excretion, representing the actual phosphate and potassium daily intake, in metabolic stable patients.

Finally, the amount of oral bicarbonate needed to maintain bicarbonate plasma levels between 24 and 28 mEq/L was evaluated every 3 months.

## 3. Statistical Analysis

Data were reported as mean ± SD or counts (percentage), when appropriate. Un-paired *T*-test and Chi-square test were used to assess differences between study groups at baseline and study completion. Two-tailed probability values ≤0.05 were considered statistically significant. Analyses were completed using R version 3.1.3 (9 March 2015) (The R Foundation for Statistical Computing).

## 4. Results

In VLPD group, the causes of renal disease were hypertensive-vascular nephropathies in 38% of patients, glomerulonephrites in 20%, tubule-interstitial nephropathies in 15%, unknown causes in 27% of patients; in control group the percentages were similar: 41%, 18%, 16%, and 24%, respectively. Moreover, the frequency of cardiovascular complications (angina, infarct, ictus) was 46% in VLPD group, and 42% in control group (*p* = NS).

Table 2 shows the differences between VLPD and control diet at baseline.

Age and percentage of male sex were not different between the two groups because of the case-control design of the study. On the other hand, VLPD patients had a lower body weight (71.6 ± 13.1 vs. 77.8 ± 14.2 kg; *p* < 0.0001). The other biochemical parameters were not different except for urinary creatinine (69.8 ± 29.1 in VLPD vs. 99 ± 32.7 µmol/day in control group; *p* < 0.0001), as consequence of different body weights and residual renal function in the two groups (26 ± 12 mL/min in VLPD group vs. 39 ± 14 mL/min in control group; *p* < 0.0001). There were no statistical differences between the two groups regarding systolic blood pressure (SBP), diastolic blood pressure (DBP), protein and phosphate intake, urinary natrium, potassium, phosphate and urea nitrogen, NEAP, and PRAL (Table 2).

Table 3 shows differences at 6 and 12 months of the same parameters seen in Table 2. VLPD patients showed at 6 and also 12 months a significant reduction of SBP (*p* < 0.0001), DBP (*p* < 0.001), plasma urea (*p* < 0.0001) protein intake (*p* < 0.0001), calcemia (*p* < 0.0001), phosphatemia (*p* < 0.0001), phosphate intake (*p* < 0.0001), urinary natrium (*p* < 0.0001), urinary potassium (*p* < 0.002), and urinary phosphate (*p* < 0.0001). At six months potassemia was higher in VLPD group than in controls (*p* < 0.001), but not at 12 months (patients were not administrated potassium binders, and the correction of hyperpotassemia at 12 months was mostly as a consequence of a physiological correction of metabolic acidosis). 

**Figure 2:**
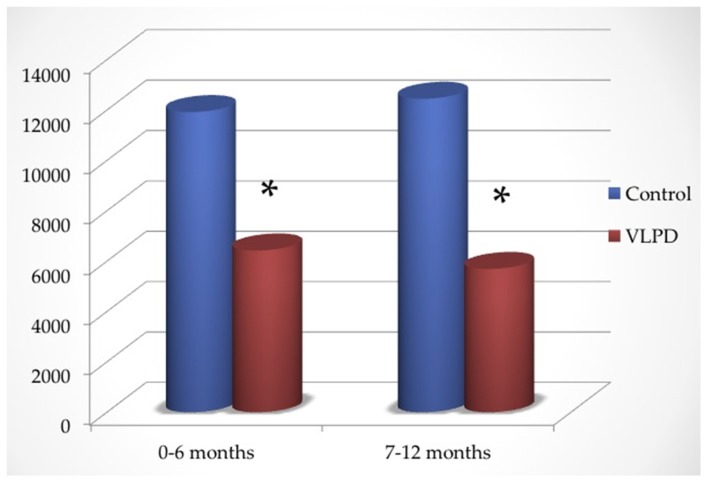
Dose of oral bicarbonate administered in control and VLPD (mmol).

As shown in Figure 2, in the first six months the dose of oral bicarbonate administered was 0.86 ± 0.31 (controls) versus 0.51 ± 0.33 mmol/kg/day (VLPD group) (*p* < 0.0001), while in the second part of follow-up it was 0.91 ± 0.42 (controls) versus 0.48 ± 0.35 mmol/kg/day (VLPD group) (*p* < 0.0001). 

Total oral bicarbonate administered in the first half of follow-up was 11,919 ± 297 mmol in controls and 6426 ± 224 mmol in VLPD patients, while in the second half of follow-up it was 12,448 ± 451 in controls and 5962 ± 374 mmol in VLPD patients (Figure 2).

Therefore, during the follow-up VLPD reduced the amount of oral bicarbonate of 30–37 mEq/day. (Table 3).

In VLPD group, NEAP dropped from 71 ± 37 mEq/day to 33 ± 16 mEq/day (after six months) and to 25 ± 11 mEq/day (after 12 months) (*p* < 0.001), while in control patients it remained unchanged (from 73 ± 35 mEq/day to 71 ± 39 mEq/day after six months and to 77 ± 41 mEq/day after 12 months (*p* = NS). Similarly, in VLPD patients PRAL reduced from 22 ± 9 mEq/day to −4.5 ± 4.1 mEq/day after six months and to −13 ± 6 mEq/day after 12 months (*p* < 0.001). It was unchanged in control patients (24 ± 13 mEq/day vs. 22 ± 9 mEq/day vs. 34 ± 11 mEq/day respectively; *p* = NS). Therefore, in VLPD patients NEAP diminished of 53% after six months (*p* < 0.0001) and of 67% after 12 months (*p* < 0.0001); PRAL decreased of 120% after six months (*p* < 0.0001) and of 138% after 12 months (*p* < 0.0001).

## 5. Discussion

Beneficial effects of a correction of metabolic acidosis has been described in several studies. In 2010, Menon showed in a post-hoc analysis of MDRD study that low plasma bicarbonate levels increased the risk of outcomes such as renal death and mortality [30]. Wesson et al. showed the paramount role of a diet rich in fruit and vegetables, not only from the nutritional point of view, but also in the nephrology field, because it ensures an amount of alkali that are needed in CKD [31,32,33,34,35,36,37,38,39,40,41].

The fact that the acid load linked to animal proteins is higher than that linked to plant proteins is already known in the scientific community [42,43,44]. Moe et al. showed that the use of only plant proteins, compared to animal proteins, was able to reduce daily serum and urinary phosphate levels in eight subjects, the load of sodium, calcium and phosphorus being equal [14]. Therefore, D’Alessandro et al.’s phosphorus pyramid seems useful as tool for dietary phosphate management [13]. Moreover, several studies showed that the use of animal proteins compared to fruit and plant proteins induced a worsening of hard outcomes such as mortality and CKD progression [39,44]; then, nutritional therapy has to be deeply analyzed, and nutritional prescription has to be focused not only on reduction of protein content but also on proteins’ quality [45].

Our study has some limitations: (1) it is a post-hoc analysis of a previous study, but with a case-control design that should reduce some bias; (2) the small number of patients (again, the case-control study has the property of amplifying the number of studied subjects); and (3) a follow-up of only 12 months.

A strength of our study is the direct measure of protein intake to calculate NEAP and PRAL with the Maroni formula based on 24-h urinary urea nitrogen instead of protein intake estimated by diary interviews, and direct measure of urinary phosphate and potassium. Our data showed that VLPD (containing high quantity of fruit and vegetables and with a very low-proteins amount supplemented with essential amino-acids and ketoanalogues of non-essential amino-acids) significantly reduced NEAP and PRAL. Essential amino-acids contained in VLPD respond to metabolic need ensuring a positive nitrogen balance, thus avoiding malnutrition, while ketoanalogues allow a significant reduction of serum urea levels using urea nitrogen to transform the ketoanalgous in the corresponding no-essential amino acid, as shown in our previous studies [46,47]. 

The importance of serum urea reduction in CKD patients, somewhat neglected in recent decades, has now also been reconsidered because of its role in the etiopathogenesis of cyanate increase [47] and of intestinal microbial flora alteration in CKD patients [48]. Finally, a higher intake of fruit and vegetables with diet has not to be considered alarming in relation to hyperpotassiemia as long as patients have a good urine output because alkali introduction (contained in fruit and vegetables) facilitates intracellular potassium shift. Moreover, there was no difference in nutritional status before and after the study in the two groups. In fact, BMI did not change in both groups: 28 ± 4 kg/m^2^ and 27 ± 4 kg/m^2^ in VLPD group, and 30 ± 5 kg/m^2^ and 29 ± 4 kg/m^2^ in controls, before and after the study, respectively. Similarly, serum albumin was also unchanged: 38 ± 7 g/L and 38 ± 3 g/L in VLPD group, and 40 ± 5 g/L and 38 ± 3 g/L in controls, respectively.

In previous studies, VLPD produced a significant decrease of proteinuria, reducing phosphate burden and renal disease progression in CKD patients, also in patients treated with angiotensin converting enzyme-inhibitors, who did not achieve a satisfying proteinuria reduction with the drug [15]. The same happened in our subjects, who showed a proteinuria reduction from 424 mg to 11 mg/day after 12 months of follow-up.

Furthermore, several studies showed that VLPD reduces oxidative stress in both experimental animals [49,50] and in humans [51].

We did not find any depressive symptoms in our patients nor in previous published studies. 

It notable that the increase of creatinine clearance from 26 to 30 mL/min after 12 months (renal function gain of 4 mL/min in one year) together with an improvement of nutritional indexes in VLPD group compared to a reduction of creatinine clearance from 39 to 30 mL/min in one year in control group (renal function loss of 9 mL/min) is suggestive of the efficacy of VLPD as nutritional therapy.

In conclusion, nutritional therapy of CKD, which has always taken into consideration a lower protein, salt, and phosphate intake, should also be adopted to correct metabolic acidosis, an important target in the treatment of CKD patients. Correction of metabolic acidosis is not only the aim of VLPD, but also of a vegetarian diet [37,38,48,52,53]. 

We provide useful indications regarding acid load of food and drinks (see Table 4A–E) that may be used as an acid load dietary “traffic light” similar to the phosphorus pyramid of D’Alessandro et al. [13]. 

The green light indicates that food can be consumed every day, while the yellow light means a two or three times a week consumption, and the red light an occasional food consumption. Of course, when using the “traffic light” information, one must always consider that the phosphorus content in plant foods have a lower absorption than that contained in foods of animal origin [12,14]; thus, animal foods with low phosphate content can lead to a higher acid load than vegetables with higher phosphate content, because of its higher intestinal absorption. Similarly, fear of potassium in fruits and vegetables in CKD is overrated, especially in CKD patients-4 and CKD-5 with retained urine flow. In fact, in these patients, hyperkalemia can be often determined by the use of aldosterone antagonists (especially for high doses) or, to a lesser degree, of inhibitors of the renin angiotensin aldosterone system drugs, or a combination of both [32,33].

Finally, nutritional therapy of CKD should take into account not only a low-protein diet, but also a rational, complex and integrated control of sodium, phosphorus and acids intake with food [18,52,54]. This means that a reduced intake of proteins is not the only important determinant of nutritional therapy of CKD but also the quality of proteins assumed and the amount of calories (no less than 30–34 kilocalories/body weight/day) in order to maintain a good metabolic and nutritional status and avoid malnutrition [55,56,57].

## Figures and Tables

**Table 1:** Diet’s composition of the two groups.

	Control	VLPD
Total Kilocalories	2320 ± 207	2110 ± 225
Total protein (g/kg/day)	0.6–1	0.3–0.4
Animal protein (%)	75	10
Vegetal protein (%)	25	90
Sodium range (mmol)	140–180	80–95
Potassium range (mmol)	40–60	30–50
Calcium range (mg)	1000–1200	500–750
Phosphorus range (mg)	700–900	300–450
Magnesium range (mg)	300–400	300–400

VLPD is a nutritional therapy used in advanced stages of CKD (from stage 3B to more severe stages) [24].

**Table 2:** Patients’ baseline data.

	All	Control	VLPD	*p*
Number	146	92	54	
Males, %	58	58	58	NS
Age, years	73.6 ± 11.2	73.7 ± 11.9	73.5 ± 10.0	NS
Height, cm	161 ± 8	162 ± 7	159 ± 8	NS
BW, kg	75.5 ± 14.1	77.8 ± 14.2	71.6 ± 13.1	0.0001
SBP, mm Hg	122 ± 20	125 ± 17	117 ± 23	NS
DBP, mm Hg	73 ± 9	74 ± 8	71 ± 10	NS
Creatinine, µmol/L	187.41 ± 68.01	180.34 ± 65.42	198.02 ± 72.49	NS
Azotemia, mmol/L	31.1 ± 11.4	31.4 ± 11.1	30.3 ± 12.1	NS
Glycaemia, mmol/L	5.6 ± 0.9	5.9 ± 0.9	5.3 ± 1.0	NS
Uric Acid, µmol/L	339.0 ± 71.4	410.4 ± 77.3	392.6 ± 59.5	NS
Natrium, mmol/L	139 ± 3	140 ± 4	139 ± 3	NS
Potassium, mmol/L	4.8 ± 1.1	4.9 ± 0.7	4.7 ± 1.3	NS
Calcium, mmol/L	2.3 ± 0.2	2.3 ± 0.7	2.2 ± 2.0	NS
Phosphorus, mmol/L	1.21 ± 0.22	1.22 ± 0.23	1.19 ± 0.20	NS
Bicarbonates, mmol/L	20.81 ± 1.72	20.77 ± 1.77	20.85 ± 1.61	NS
Cholesterol, mmol/L	3.99 ± 0.91	4.04 ± 0.85	3.94 ± 0.93	NS
Triglycerides, mmol/L	1.51 ± 0.65	1.54 ± 0.7	1.48 ± 0.58	NS
PTH, pmol/L	12.94 ± 8.81	11.88 ± 7.43	14.43 ± 10.29	NS
Haemoglobin, g/L	123 ± 17	124 ± 17	121 ± 18	NS
Albumin, g/L	39 ± 4	40 ± 5	38 ± 7	NS
CRP, mg/L	11 ± 28	10 ± 14	13 ± 35	NS
Urinary natrium, mmol/day	144 ± 78	162 ± 77	135 ± 99	NS
Urinary potassium, mmol/day	46 ± 24	52 ± 22	38 ± 19	NS
Urinary phosphate, mmol/day	197.7 ± 86.9	201.2 ± 84.3	194.4 ± 73	NS
UUN, mmol/day	7.5 ± 2.5	7.1 ± 1.8	7.8 ± 2.1	NS
Urinary proteins, g/day	0.525 ± 0.829	0.593 ± 1	0.424 ± 0.475	NS
Urinary creatinine, µmol/day	87.5 ± 34.5	99 ± 32.7	69.8 ± 29.1	0.0001
Creatinine clearance, mL/min	33 ± 15	39 ± 14	26 ± 12	0.0001
Protein intake, g/day	78 ± 10	80 ± 12	75 ± 14	NS
Phosphorus intake, mmol/day	329.5 ± 144.7	335.3 ± 140.5	324 ± 121.8	NS
NEAP	72 ± 36	73 ± 35	71 ± 37	NS
PRAL	23 ± 11	24 ± 13	22 ± 9	NS

BW: body weight; SBP: Systolic blood pressure; DBP: diastolic blood pressure; PTH: parathyroid hormone; CRP: C-reactive protein; UUN: urinary urea nitrogen; NEAP: net endogenous acid production; PRAL: potential renal acid load; NS: not significant.

**Table 3:** Data at 6 and 12 months in control group and VLPD group.

	Control 6 Months	VLPD 6 Months	*p*	Control 12 Months	VLPD 12 Months	*p*
BW, kg	77.3 ± 13.7	70.2 ± 14.1	0.003	76.0 ± 13.0	69.1 ± 14.5	0.0001
SBP, mmHg	124 ± 17	115 ± 14	0.0001	128 ± 17	110 ± 16	0.0001
DBP, mmHg	76 ± 7	71 ± 8	0.0001	76 ± 9	70 ± 8	0.0001
Creatinine, µmol/L	192.71 ± 66.3	194.48 ± 74.26	NS	196.25 ± 62.76	195.36 ± 71.6	NS
Azotemia, mmol/L	31.06 ± 11.78	16.8 ± 8.6	0.0001	32.1 ± 11.1	14.6 ± 7.8	0.0001
Glycaemia, mmol/L	6.5 ± 1.4	6.0 ± 1	NS	6.3 ± 1.2	6.2 ± 0.9	NS
Uric acid, µmol/L	327.1 ± 118.9	315.2 ± 107.1	NS	285.5 ± 95.2	291.4 ± 29.7	NS
Natrium, mmol/L	139 ± 4	139 ± 3	NS	140 ± 3	137 ± 3	NS
Potassium, mmol/L	4.7 ± 0.5	5.3 ± 0.5	0.0001	4.8 ± 0.6	4.9 ± 0.5	NS
Calcium, mmol/L	2.3 ± 0.1	2.1 ± 0.3	0.0001	2.3 ± 0.1	2.2 ± 0.2	0.0001
Phosphate, mmol/L	1.4 ± 0.4	1.2 ± 0.2	0.0001	1.5 ± 0.4	1.1 ± 0.2	0.0001
Bicarbonates, mmol/L	25.85 ± 1.90	26.81 ± 1.80	NS	25.71 ± 1.52	26.13 ± 1.66	NS
Cholesterol, mmol/L	4.1 ± 0.8	3.9 ± 1	NS	4.4 ± 0.8	3.8 ± 0.8	NS
Triglycerides, mmol/L	1.6 ± 0.8	1.5 ± 0.7	NS	1.4 ± 0.6	1.2 ± 0.5	NS
PTH, pmol/L	14.7 ± 15.9	12.2 ± 8.4	NS	22.4 ± 9.6	13.1 ± 8.7	0.0001
Hb, g/L	123 ± 15	116 ± 20	NS	123 ± 15	124 ± 10	NS
Albumin, g/L	38 ± 4	38 ± 5	NS	38 ± 4	38 ± 3	NS
CRP, mg/L	7 ± 10	6 ± 15	NS	6 ± 8	5 ± 10	NS
Urinary natrium, mmol/day	142 ± 61	99 ± 36	0.0001	142 ± 58	95 ± 36	0.0001
Urinary potassium, mmol/day	46 ± 18	31 ± 21	0.002	39 ± 13	47 ± 20	0.004
Urinary phosphate, mmol/day	191.2 ± 76.9	112.7 ± 51.7	0.0001	76.9 ± 90.4	97.9 ± 61.4	0.0001
UUN, mmol/day	6.1 ± 1.8	2.9 ± 0.7	NS	6.4 ± 2.5	2.9 ± 1.1	0.0001
Urinary proteins, g/day	0.429 ± 0.45	0.382 ± 0.42	NS	0.452 ± 0.661	0.211 ± 0.161	0.009
Urinary creatinine, µmol/day	86.6 ± 30	81.3 ± 27.4	NS	88.4 ± 3.5	80.4 ± 25.6	NS
Creatinine Clearance, mL/min	37 ± 15	28 ± 14	0.0001	35 ± 14	30 ± 17	NS
Protein intake, g/day	77 ± 14	25 ± 7	0.0001	81 ± 16	30 ± 17	0.0011
Phosphorus intake, mmol/day	318.8 ± 128.2	155.7 ± 86.2	0.0001	238.4 ± 93.7	117.5 ± 78.2	0.0001
NEAP	71 ± 39	33 ± 16	0.0001	77 ± 41	25 ± 11	0.0001
PRAL	22 ± 9	−4.5 ± 4.1	0.0001	34 ± 11	−13 ± 6	0.0001

BW: body weight; SBP: Systolic blood pressure; DBP: diastolic blood pressure; PTH: parathyroid hormone; CRP: C-reactive protein; UUN: urinary urea nitrogen. NS: not significant.

**Table:** **(A)**

	PRAL	K	P
**BEVERAGES**			
Apple juice	−2.2	101	5
Beetroot juice	−3.9		
Carrot juice	−4.8	292	42
Espresso	−2.3	115	3
Lemon juice	−2.5	140	10
Orange juice	−2.9	200	17
Red wine	−2.4	127	23
Tomato juice	−2.8	188	19
Vegetable	−3.6	193	17
Apple vinegar	−2.3		8
**VEGETABLE**			
Huzelnuts	−2.8	466	322
Apples	−2.2	132	12
Apricots	−4.8	320	16
Bananas	−5.5	350	28
Black currants	−6.5	370	43
Cherries	−3.6	229	18
Figs, dried	−18.1	1010	111
Grapes	−3.9	192	4
Kiwi	−4.1	400	34
Lemon	−2.6	140	11
Mango	−3.3	250	14
Orange	−2.7	200	22
Peache	−2.4	260	20
Pear	−2.9	127	11
Pineapple	−2.7	250	8
Raisins	−21	275	29
Strawberries	−2.2	160	28
Watermelon	−1.9	280	25
Broussel sprouts	−4.5	450	50
Carrots	−4.9	220	37
Cauliflower	−4.0	350	69
Celery	−5.2	280	45
Chicory	−2.0	180	31
Eggplant	−3.4	184	33
Fennel	−7.9	394	39
Kale	−7.8	243	29
Kohlrabi	−5.5	191	29
Lamb’s Lettuce	−5.0	459	30
Lettuce	−2.5	240	31
Potatoes	−4.0	570	54
Radish, red	−3.7	240	75
Ruccola	−7.5	468	52
Sauerkraut	−3.0	170	20
Soy beans	−3.4	485	142
Spinach	−14.0	530	62
Tomato	−3.1	297	26
Zucchini	−4.6	264	65
Brans, green	−3.1	566	734
Parsley	−12.0	670	75
Basil	−7.3	300	56
Chives	−5.3	296	48

**Table:** **(B)**

	PRAL	K	P
**BEVERAGES**			
Mineral water	−0.1		
Beer, draft	−0.2	27	14
Beer, stout	−0.1	27	14
White wine	−1.2	71	74
Cocoa, made with semi-skimmed milk	−0.4	169	105
Coffee, infusion	−1.4	30	3
Fruit tea, infusion	−0.3	19	36
Grape juice	−1.0	88	4
Green tea, infusion	−0.3	20	
Herb tea	−0.2	21	0
Tea, Indian, infusion	−0.3		
Wine vinegar	−1.6	39	8
**FAT and OIL**			
Margarine	−0.5	18	5
Olive oil	0.0	1	0
Sunflower seed oil	0.0	0	0
**VEGETABLES**			
Asparagus	−0.4	172	65
Broccoli, green	−1.2	316	66
Cucumber	−0.8	140	17
Garlic	−1.7	401	153
Gherkin, pickled	−1.6		
Leeks	−1.8	180	35
Lettuce, iceberg	−1.6	141	20
Mushrooms	−1.6	318	86
Onions	−1.5	146	29
Peppers, Capsicum, green	−1.4	1260	158
Soy milk	−0.8	143	
Tofu	−0.8	151	52
Whey	−1.6	143	78
**SWEATS**			
Honey	−0.3	52	6
Ice cream, fruit, mixed	−0.6	188	100
Marmalade	−1.5	77	19
Nougat hazelnut cream	−1.4	407	152
Sugar, brown	−1.2	133	4
Sugar, white	0.0	2	

**Table:** **(C)**

	PRAL	K	P
**BEVERAGES**			
Coca cola	0.4	20	24.8
**FAT and OIL**			
Butter	0.6	15	16
**VEGETABLE**			
Lentils, green and brown, whole, dried	3.5	980	347
Peas	1.2	990	320
Buttermilk	0.5	145	93
Cream, fresh, sour	1.2	119	66
Curd cheese	0.9	118	180
Egg, white	1.1	163	15
Milk, whole, evaporated	1.1	390	23.5
Milk, whole, pasteurized and sterilized	0.7	132	84
Skimmed milk	0.7	156	84
Yogurt, whole milk, fruit	1.2	177	109
Yogurt, whole milk, plain	1.5	155	95
**CEREALS, FLOUR and PASTA**			
Rice, white, boiled	1.7	56	55
Bread, wheat flour, whole meal	1.8	110	103
Chocolate, bitter	0.4	300	147
Chocolate, milk	2.4	420	208
Ice cream, dairy vanilla	0.6	199	105

**Table:** **(D)**

	PRAL	K	P
**VEGETABLES**			
Peanuts, plain	8.3	680	290
Pistachio	8.5	1025	490
Sweet almond	4.3	780	484
Walnuts	6.8	441	346
**ANIMAL FOOD**			
Carp	7.9	333	415
Cod, fillets	7.1	235	281
Haddock	6.8	286	227
Halibut	7.8	435	236
Herring	7.0	419	303
Rose-fish	10.0	269	200
Salmon	9.4	505	371
Salted matie (herring)	8.0	447	325
Shrimps	7.6	89	282
Sole	7.4	160	252
Zander	7.1	269	200
Beaf, lean only	7.8	349	211
Cervelat sausage	8.9	260	190
Chicken	8.7	229	194
Duck	4.1	230	269
Lamb, lean only	7.6	318	191
Pork sausage	7.0	260	192
Rump steak, lean and fat	8.8	221	356
Turkey, meat only	9.9	275	186
Veal, fillet	9.0	325	212
Cottage cheese, plain	8.7		
Egg, chicken, whole	8.2	138	198
Full-fat soft cheese	4.3		
**CEREALS, FLOUR and PASTA**			
Amaranth	7.5	508	557
Barely (whole meal)	5.0	75	40
Bread, rye flour	4.1	166	125
Bread, white wheat	3.7	110	103
Buckwheat (whole grain)	3.7	460	330
Coarse whole meal bread	5.3		
Cornflakes	6.0	73	49
Crisp bread, rye	3.3	183	138
Macaroni	6.1	20	32
Noodles	6.4	22	20
Pumpernickel	4.2	208	178
Rice, white	4.6	75	96
Rusk	5.9	224	430
Rye, flour	4.4	510	332
Spaetzle (German sort)	9.4	38	
Spaghetti	6.5	44	58
Spaghetti, whole meal	7.3	44	89
Wheat flour, whole meal	8.2	337	300
Whole meal bread	7.2	248	202

**Table:** **(E)**

	PRAL	K	P
**ANIMAL FOOD**			
Eel, smoked	11,0	349	277
Mussels	15.3	268	236
Prawn	15.5	89	282
Sardines in oil	13.5	397	490
Tiger Prawn	18.2	170	306
Trout, steamed	10.8	361	220
Corned beef, canned	13.2	402	125
Goose, lean only	13.0	388	309
Liver (veal)	14.2	308	350
Luncheon meat, canned	15.4	564	109
Ox liver fegato bue	15.4	320	350
Pig’s liver	15.7	273	362
Rabbit, lean only	19.0	383	180
Salami	11.6	452	225
Camembert	14.6	100	347
Cheddar-type, reduced fat	26.4	120	545
Edam cheese full fat	19.4	188	536
Egg, yolk	23.4	90	586
Emmental cheese full fat	21.1	107	700
Fresh cheese (Quark)	11.1	150	650
Gouda	18.6	121	443
Hard cheese	19.2	55	675
Parmesan	34.2	102	800
Processed cheese, plain	28.7		943
Rich cream full fat cheese	13.2	284	
**CEREALS, FLOUR and PASTA**			
Oat flakes	10.7	429	523
Rice, brown	12.5	223	333

The green light indicates that food can be consumed every day, while the yellow light means a two or three times a week consumption, and the red light an occasional food consumption

## References

[1] Kopple J.D. (2000). National Kidney Foundation: K/DOQI clinical practice guidelines for nutrition in chronic renal failure. Am. J. Kidney Dis..

[2] Chen W., Abramowitz M.K. (2014). Metabolic acidosis and the progression of chronic kidney disease. BMC Nephrol..

[3] Huston H.K., Abramowitz M.K., Zhang Y., Greene T., Raphael K.L. (2014). Net endogenous acid production and mortality in NHANES III. Nephrology (Carlton).

[4] Kobayashi S., Maesato K., Moriya H., Ohtake T., Ikeda T. (2005). Insulin resistance in patients with chronic kidney disease. Am. J. Kidney Dis..

[5] Mak R.H.K. (1998). Effect of metabolic acidosis on insulin action and secretion in uremia. Kidney Int..

[6] Kurella M., Lo J.C., Chertow G.M. (2005). Metabolic syndrome and the risk for chronic kidney disease among nondiabetic adults. J. Am. Soc. Nephrol..

[7] De Brito-Ashurst I., Varagunam M., Raftery M.J., Yaqoob M.M. (2009). Bicarbonate supplementation slows progression of CKD and improves nutritional status. J. Am. Soc. Nephrol..

[8] Mahajan A., Simoni J., Sheather S.J., Broglio K.R., Rajab M.H., Wesson D.E. (2010). Daily oral sodium bicarbonate preserves glomerular filtration rate by slowing its decline in early hypertensive nephropathy. Kidney Int..

[9] Goraya N., Simoni J., Jo C.H., Wesson D.E. (2014). Treatment of metabolic acidosis in patients with stage 3 chronic kidney disease with fruits and vegetables or oral bicarbonate reduces urine angiotensinogen and preserves glomerular filtration rate. Kidney Int..

[10] Goraya N., Wesson D.E. (2014). Is dietary acid a modifiable risk factor for nephropathy progression?. Am. J. Nephrol..

[11] Appel L.J., Moore T.J., Obarzanek E., Vollmer W.M., Svetkey L.P., Sacks F.M., Bray G.A., Vogt T.M., Cutler J.A., Windhauser M.M. (1997). A clinical trial of the effects of dietary patterns on blood pressure. DASH Collaborative Research Group. N. Engl. J. Med..

[12] Bellizzi V., Di Iorio B.R., De Nicola L., Minutolo R., Zamboli P., Trucillo P., Catapano F., Cristofano C., Scalfi L., Conte G. (2007). Very low protein diet supplemented with ketoanalogs improves blood pressure control in chronic kidney disease. Kidney Int..

[13] D’Alessandro C., Piccoli G.B., Cupisti A. (2015). The “phosphorus pyramid”: A visual tool for dietary phosphate management in dialysis and CKD patients. BMC Nephrol..

[14] Moe S.M., Zidehsarai M.P., Chambers M.A., Jackman L.A., Radcliffe J.S., Trevino L.L., Donahue S.E., Asplin J.R. (2011). Vegetarian compared with meat dietary protein source and phosphorus homeostasis in chronic kidney disease. Clin. J. Am. Soc. Nephrol..

[15] Di Iorio B.R., Bellizzi V., Bellasi A., Torraca S., D’Arrigo G., Tripepi G., Zoccali C. (2013). Phosphate attenuates the anti-proteinuric effect of very low-protein diet in CKD patients. Nephrol. Dial. Transplant..

[16] Zoccali C., Ruggenenti P., Perna A., Leonardis D., Tripepi R., Tripepi G., Mallamaci F., Remuzzi G., REIN Study Group (2011). Phosphate may promote CKD progression and attenuate renoprotective effect of ACE inhibition. J. Am. Soc. Nephrol..

[17] Brunori G., Viola B.F., Parrinello G., De Biase V., Como G., Franco V., Garibotto G., Zubani R., Cancarini G.C. (2007). Efficacy and safety of a very-low-protein diet when postponing dialysis in the elderly: A prospective ran- domized multicenter controlled study. Am. J. Kidney Dis..

[18] Fouque D., Mitch W.E. (2015). Low-protein diets in chronic kidney disease: Are we finally reaching a consensus?. Nephrol. Dial. Transplant..

[19] Bellizzi V., Chiodini P., Cupisti A., Viola B.F., Pezzotta M., De Nicola L., Minutolo R., Barsotti G., Piccoli G.B., di Iorio B. (2015). Very low-protein diwt plus ketoacids in CKD and risk of death during ESRD: A historical cohort controlled study. Nephrol. Dial. Transplant..

[20] Chauveau P., Fouque D., Combe C., Aparicio M. (2013). Evolution of the diet from the paleolithic to today: Progress or regress?. Nephrol. Ther..

[21] Adeva M.M., Souto G. (2011). Diet-induced metabolic acidosis. Clin. Nutr..

[22] Ikizler H.O., Zelnick L., Ruzinskin J., Curtin L., Utzschneider K.M., Kestenbaum B., Himmelfarb J., De Boer I.H. (2016). Dietary acid load is associated with serum bicarbonate but not insulin sensivity in CKD. J. Ren. Nutr..

[23] Di Iorio B., Aucella F., Conte G., Cupisti A., Santoro D. (2012). A prospective, multicenter, randomized, controlled study: The correction of metabolic acidosis with Use of Bicarbonate in Chronic Renal Insufficiency (UBI) Study. J. Nephrol..

[24] Aparicio M., Bellizzi V., Chauveau P., Cupisti A., Ecder T., Fouque D., Garneata L., Lin S., Mitch W.E., Teplan V. (2012). Keto-acid therapy in predialysis chronic kidney disease patients: Final consensus. J. Ren. Nutr..

[25] Di Iorio B., de Simone E., Quintaliani P. (2016). Protein Intake with diet or nutritional therapy in ESRD. A different point of view for non specialists. G. Ital. Nefrol..

[26] Maroni B.J., Steinman T.I., Mitch W.E. (1985). A method for estimating nitrogen intake of patients with chronic renal failure. Kidney Int..

[27] Frassetto L.A., Lanham-New S.A., MacDonald H.M., Remer T., Sebastian A., Tucker K.L., Tylavsky F.A. (2007). Standardizing terminology for estimating the diet-dependent net acid load to the metabolic system. J. Nutr..

[28] Remer T., Manz F. (1994). Estimation of the renal net acid excretion by adults consuming diets containing variable amounts of protein. Am. J. Clin. Nutr..

[29] Frassetto L.A., Todd K.M., Morris R.J.C., Sebastian A. (1998). Estimation of net endogenous noncarbonic acid production in humans from diet potassium and protein contents. Am. J. Clin. Nutr..

[30] Menon V., Tighiouart H., Vaughn N.S., Beck G.J., Kusek J.W., Collins A.J., Greene T., Sarnak M.J. (2010). Serum bicarbonate and long-term outcomes in CKD. Am. J. Kidney Dis..

[31] Phisitkul S., Khanna A., Simoni J., Broglio K., Sheather S., Rajab M.H., Wesson D.E. (2010). Amelioration of metabolic acidosis in patients with low GFR reduced kidney endothelin production and kidney injury, and better preserved GFR. Kidney Int..

[32] Wesson D.E., Jo C.H., Simoni J. (2015). Angiotensin II-mediated GFR decline in subtotal nephrectomy is due to acid retention associated with reduced GFR. Nephrol. Dial. Transplant..

[33] Wesson D.E., Simoni J. (2010). Acid retention during kidney failure induces endothelin and aldosterone production which lead to progressive GFR decline, a situation ameliorated by alkali diet. Kidney Int..

[34] Wesson D.E., Simoni J., Broglio K., Sheather S. (2011). Acid retention accompanies reduced GFR in humans and increases plasma levels of endothelin and aldosterone. Am. J. Physiol. Ren. Physiol..

[35] Goraya N., Wesson D.E. (2013). Does correction of metabolic acidosis slow chronic kidney disease progression?. Curr. Opin. Nephrol. Hypertens..

[36] Goraya N., Wesson D.E. (2012). Acid-base status and progression of chronic kidney disease. Curr. Opin. Nephrol. Hypertens..

[37] Wesson D.E., Jo C.H., Simoni J. (2012). Angiotensin II receptors mediate increased distal nephron acidification caused by acid retention. Kidney Int..

[38] Goraya N., Wesson D.E. (2015). Dietary interventions to improve outcomes in chronic kidney disease. Curr. Opin. Nephrol. Hypertens..

[39] Goraya N., Simoni J., Jo C.H., Wesson D.E. (2013). A comparison of treating metabolic acidosis in CKD stage 4 hypertensive kidney disease with fruits and vegetables or sodium bicarbonate. Clin. J. Am. Soc. Nephrol..

[40] Goraya N., Wesson D.E. (2016). Dietary Protein as Kidney Protection: Quality or Quantity?. J. Am. Soc. Nephrol..

[41] Phisitkul S., Hacker C., Simoni J., Tran R.M., Wesson D.E. (2008). Dietary protein causes a decline in the glomerular filtration rate of the remnant kidney mediated by metabolic acidosis and endothelin receptors. Kidney Int..

[42] Banerjee T., Crews D.C., Wesson D.E., Tilea A.M., Saran R., Ríos-Burrows N., Williams D.E., Powe N.R., Centers for Disease Control and Prevention Chronic Kidney Disease Surveillance Team (2015). High Dietary Acid Load Predicts ESRD among Adults with CKD. J. Am. Soc. Nephrol..

[43] Wesson D.E., Nathan T., Rose T., Simoni J., Tran R.M. (2007). Dietary protein induces endothelin-mediated kidney injury through enhanced intrinsic acid production. Kidney Int..

[44] Gutiérrez O.M., Muntner P., Rizk D.V., McClellan W.M., Warnock D.G., Newby P.K., Judd S.E. (2014). Dietary patterns and risk of death and progression to ESRD in individuals with CKD: A cohort study. Am. J. Kidney Dis..

[45] Di Iorio B.R., Minutolo R., De Nicola L., Bellizzi V., Catapano F., Iodice C., Rubino R., Conte G. (2003). Supplemented very low protein diet ameliorates responsiveness to erythropoietin in chronic renal failure. Kidney Int..

[46] Bellizzi V., Cupisti A., Locatelli F., Bolasco P., Brunori G., Cancarini G., Caria S., De Nicola L., Di Iorio B.R., Di Micco L. (2016). Low-protein diets for chronic kidney disease patients: The Italian experience. BMC Nephrol..

[47] El-Gamal D., Rao S.P., Holzer M., Hallström S., Haybaeck J., Gauster M., Wadsack C., Kozina A., Frank S., Schicho R. (2014). The urea decomposition product cyanate promotes endothelial dysfunction. Kidney Int..

[48] Vaziri N.D., Wong J., Pahl M., Piceno Y.M., Yuan J., DeSantis T.Z., Ni Z., Nguyen T.H., Andersen G.L. (2013). Chronic kidney disease alters intestinal microbial flora. Kidney Int..

[49] Gao X., Wu J., Dong Z., Hua C., Hu H., Mei C. (2010). A low-protein diet supplemented with ketoacids plays a more protective role against oxidative stress of rat kidney tissue with 5/6 nephrectomy than a low-protein diet alone. Br. J. Nutr..

[50] Gao X., Huang L., Grosjean F., Esposito V., Wu J., Fu L., Hu H., Tan J., He C., Gray S., Jain M.K. (2011). Low-protein diet supplemented with ketoacids reduces the severity of renal disease in 5/6 nephrectomized rats: A role for KLF15. Kidney Int..

[51] Di Iorio B.R., Cucciniello E., Martino R., Frallicciardi A., Tortoriello R., Struzziero G. (2009). Acute and persistent antiproteinuric effect of a low-protein diet in chronic kidney disease. G Ital. Nefrol..

[52] Garneata L., Stancu A., Dragomir D., Stefan G., Mircescu G. (2016). Ketoanalogue-Supplemented Vegetarian Very Low-Protein Diet and CKD Progression. J. Am. Soc. Nephrol..

[53] Chauveau P., Combe C., Fouque D., Aparicio M. (2013). Vegetarianism: Advantages and drawbacks in patients with chronic kidney diseases. J. Ren. Nutr..

[54] Piccoli G.B., Ferraresi M., Deagostini M.C., Vigotti F.N., Consiglio V., Scognamiglio S., Moro I., Clari R., Fassio F., Biolcati M. (2013). Vegetarian low-protein diets supplemented with keto analogues: A niche for the few or an option for many?. Nephrol. Dial. Transplant..

[55] Fouque D., Chen J., Chen W., Garneata L., Hwang S.J., Kalantar-Zadeh K., Kopple J.D., Mitch W.E., Piccoli G., Teplan V. (2016). Adherence to ketoacids/essential amino acids-suppleented low protein diets and new indications for patients with chronic kidney disease. BMC Nephrol..

[56] Aparicio M., Bellizzi V., Chauveau P., Cupisti A., Ecder T., Fouque D., Garneata L., Lin S., Mitch W., Teplan V. (2013). Do ketoanalogues still have a role in delaying dialysis initiation in CKD predialysis patients?. Semin. Dial..

[57] Mitch W.E., Remuzzi G. (2016). Diets for patients with chronic kidney disease, should we reconsider?. BMC Nephrol..

